# Exploring Health-Seeking Behavior Among Women Diagnosed With Gestational Diabetes Mellitus: A Qualitative Study

**DOI:** 10.7759/cureus.112385

**Published:** 2026-07-10

**Authors:** Aditi Das, Kalyani Rath, Swagata Sahoo, Mohini

**Affiliations:** 1 Obstetrics and Gynaecology, Kalinga Institute of Nursing Sciences, Kalinga Institute of Industrial Technology (KIIT) Deemed to be University, Bhubaneswar, IND

**Keywords:** gestational diabetes mellitus, health-seeking behavior, health-seeking behaviour, patient-centered care, qualitative study, women

## Abstract

Background

Gestational diabetes mellitus (GDM) constitutes a significant public health challenge in India, particularly impacting pregnant women and posing risks to both maternal and fetal health. Despite the growing prevalence of GDM, limited qualitative evidence is available regarding the health-seeking behaviors of women in Odisha. This study aimed to explore the health-seeking behaviors among women diagnosed with GDM.

Methods

A descriptive exploratory research design was used at a tertiary care hospital in Odisha, India. Women diagnosed with GDM were recruited through purposive sampling, excluding those with pre-existing chronic conditions. One-on-one interviews were conducted with 21 participants in the Odia language, audio-recorded, transcribed verbatim, and analyzed using thematic analysis supported by NVivo software, version 14 (trial version; Lumivero, Denver, CO, USA). Trustworthiness was ensured through member checking, prolonged engagement, and detailed contextual descriptions.

Results

Thematic analysis was performed for the transcripts, and six key themes emerged from the analysis: emotional responses to diagnosis, including fear, guilt, and denial; inconsistent information delivery from healthcare providers; expectations for empathetic care, continuity of care, and peer support; positive changes in health behaviors, such as improved dietary practices, increased physical activity, and heightened health awareness; barriers to healthcare access, which included transportation challenges, financial constraints, and cultural or linguistic obstacles; and determinants of health-seeking behavior shaped by family support, cultural beliefs, competing responsibilities, and perceptions of medical interventions. Family encouragement was found to facilitate adherence, while misconceptions and logistical barriers hindered effective management.

Conclusion

This study identified key themes surrounding health-seeking behaviors, specifically emotional responses to diagnosis, the perceived adequacy of information, expectations of healthcare providers, positive lifestyle modifications, barriers to accessing care, and the overarching determinants of health-seeking behavior. To optimize patient outcomes, these dimensions must be actively addressed by healthcare personnel and systematically integrated into healthcare services.

## Introduction

Diabetes is largely influenced by lifestyle factors and has increased at an alarming rate across all age groups in India, particularly among younger people, where the prevalence has crossed 10%. India is now often referred to as the “Diabetes Capital of the World” [1], accounting for nearly 17% of global diabetes cases. This condition has emerged as a major public health concern, with almost 80 million people currently affected in India, and the number is projected to rise to 135 million by 2045 [2].

Gestational diabetes mellitus (GDM) refers to a form of hyperglycemia or glucose intolerance that is first identified during pregnancy. It is usually characterized by blood glucose levels that are higher than normal pregnancy thresholds but do not meet the criteria for overt diabetes. GDM is the most common medical complication during pregnancy worldwide and poses risks to both the mother and the fetus [3].

The global prevalence of GDM remains high. According to the 2021 International Diabetes Federation (IDF) Diabetes Atlas, about 14.0% of pregnancies, nearly one in seven, are affected. The burden varies considerably across regions, with the highest prevalence reported in the Middle East and North Africa (27.6%) and South-East Asia (20.8%) [4]. Overall, diabetes during pregnancy affects around one in six live births, amounting to nearly 21 million cases each year [5]. In India, the prevalence of GDM is about 22.4%, which means roughly one in four pregnant women is affected. Regional variation is notable: central India reports the highest prevalence at 32.9%, followed by north India at 31.4%, south India at 24.2%, east India at 17.9%, northeast India at 16.6%, and western India at the lowest at 16%. Urban areas show a slightly higher prevalence (24.2%) than rural areas (21.6%), although the difference is small [6, 7]. In Odisha, GDM prevalence among pregnant women ranges from 9.89% to 15.9%, while one study in Cuttack reported a prevalence of 57.1% among sedentary women. Factors such as rapid urbanization, a shift toward calorie-dense processed foods, low levels of physical activity, and genetic susceptibility may contribute to this pattern. These findings underline the need for targeted screening and lifestyle interventions for high-risk groups across the state [8].

GDM raises the risk of maternal complications such as preeclampsia, cesarean delivery, and future type 2 diabetes. It is also associated with neonatal complications, including macrosomia, hypoglycemia, and long-term metabolic disorders [9]. Effective management depends greatly on timely health-seeking behavior, which includes recognizing symptoms, utilizing healthcare services, adopting self-management practices such as diet and exercise, and attending postpartum screening. However, global barriers such as poor health literacy and limited access to care often result in suboptimal health-seeking behavior [10, 11].

Health-seeking behavior among women with GDM is influenced by several factors, including sociodemographic characteristics such as age and income; cultural influences like traditional remedies and stigma; psychological aspects such as self-efficacy and fear; and access to healthcare services. These often contribute to delayed diagnoses or poor treatment adherence [12, 13]. In resource-limited settings such as northern Nigeria, studies have reported low antenatal attendance, dependence on family and spiritual support, and non-compliance with treatment. Although many women are aware of prescribed regimens, only 70% adhere to them [14]. Qualitative findings also point to emotional distress, misunderstandings about the temporary nature of GDM, and the role of online peer communities in filling gaps in formal care by offering both informational and emotional support [15, 16]. Migrant women and elderly primigravida women face additional challenges, including a shift in beliefs from concern about fetal risk during pregnancy to personal health risk after childbirth [17]. Although research on GDM has generated substantial quantitative evidence, qualitative insights into health-seeking behaviors remain limited. This points to the need for deeper exploration of the barriers and facilitators that can inform targeted interventions and improve maternal and fetal outcomes [18, 19].

This study explores the health-seeking behaviors of women with GDM, with particular attention to the social, cultural, and psychological barriers that affect adherence to medical advice. It also examines how environmental factors and support systems shape self-care during and after pregnancy. By using a qualitative approach, the study seeks to bridge the gap between clinical guidelines and the real-life challenges women face, ultimately helping healthcare providers deliver more patient-centered care.

## Materials and methods

Study design

A descriptive exploratory research design was used to investigate the health-seeking behaviours of women diagnosed with GDM.

Sample and sampling strategy

The study was conducted in the outpatient Department of Obstetrics and Gynaecology at Pradyumna Bal Memorial Hospital (Kalinga Institute of Industrial Technology), a selected tertiary care hospital in Bhubaneswar, India, between December 2025 and February 2026. A total of 21 women between 33 and 37 weeks of gestation diagnosed with GDM were included. Participants were recruited using a purposive sampling technique from the population that met the predetermined inclusion criteria, comprising primigravida women between 33 and 37 weeks of gestation diagnosed with GDM who provided informed consent and attended the outpatient Department of Obstetrics and Gynecology. Women were excluded if they had a previous diagnosis of chronic diseases such as cardiovascular disease or kidney disease, a history of hypertension, or known pre-existing diabetes.

Data collection

Data collection was carried out after obtaining Institutional Ethics Committee (IEC) approval and permission from the medical superintendent. The purpose of the study was clearly communicated to the participants, and written informed consent was obtained from the participating women. A semi-structured interview guide, developed through a review of existing literature and consultation with subject experts, was used to explore participants’ health-seeking behaviors (Appendix A). The approximate duration of data collection was communicated to them. Data were collected through one-on-one in-depth interviews from December 2025 to February 2026 in the examination room near the antenatal outpatient department. A total of 21 primigravida women with gestational weeks between 33 and 37 diagnosed with GDM were interviewed for this study. All interviews were conducted in the Odia language to ensure clarity and ease of communication. Participants were approached when they were physically and emotionally comfortable. Each interview lasted approximately 30 to 40 minutes and was audio-recorded with the participants' informed consent. All interviews were transcribed verbatim, cross-checked with audio recordings for accuracy, and anonymized to ensure confidentiality. Member checking was conducted the following day to confirm and clarify responses, and gratitude was expressed to all participants for their contributions.

Data analysis

Thematic content analysis was carried out, starting with an immersive review of the transcripts through multiple readings to ensure a thorough understanding of the data. This was followed by systematic coding to identify significant features within the dataset. Related codes were then organized and clustered into preliminary themes, capturing salient patterns that effectively reflected participants’ experiences and perceptions in a coherent and meaningful way. NVivo software, version 14 (trial version; Lumivero, Denver, CO, USA), was employed to aid in the organization, coding, and retrieval of data, thus enhancing the rigor and transparency of the analytic process.

Trustworthiness of the study (rigor)

The trustworthiness of this qualitative study on health-seeking behavior among women with GDM was ensured by following the four key criteria: credibility, transferability, dependability, and confirmability. Credibility was strengthened through prolonged engagement in in-depth, semi-structured interviews, which were audio-recorded to capture participants’ responses accurately. Member checking was conducted the following day to verify the transcriptions and interpretations, while field notes were used to record non-verbal cues, contextual details, and other observational insights. Dependability was maintained through careful documentation of the data collection process, the use of code-recode procedures, and peer examination. In addition, interviews were audio-recorded and transcribed verbatim, and these transcripts were reviewed by language experts. Transferability was supported through purposive sampling. Data saturation was reached after 18 interviews and extended to 21 to gain deeper insights. Verbatim transcripts, field notes, and participant quotations further added contextual richness. Confirmability was established through peer debriefing and inquiry audits, during which the raw data, coding, and interpretations were reviewed. Mobile debriefing sessions with mothers, close examination of transcripts, and independent coding until consensus was reached also helped ensure confirmability.

## Results

A total of 21 women diagnosed with GDM participated in a one-to-one interview. The sociodemographic and clinical profile of participants with GDM revealed that the mean age was 30.29 ± 2.97 years and the mean gestational age was 35 ± 1.34 weeks. The mean gestational age at diagnosis was 17.6 ± 3.6 weeks, and the mean blood glucose level was 123.5 ± 6.08 mg/dL. Most participants were college-educated (42.9%), employed (57.1%), and belonged to the middle socioeconomic group. The majority were Hindu (66.7%). A considerable proportion had a family history of diabetes, mainly maternal (42.9%). Most participants were managed with combination therapy (38.1%), followed by medical nutrition therapy (33.3%) (Table 1).

**Table 1:** Sociodemographic Characteristics of Women With Gestational Diabetes Mellitus (n=21) ₹: indian rupee

Serial Number	Socio-demographic Variables	Mean ± SD	
1	Age, in Years	30.29 ± 2.97	
2.	Gestational Age (Weeks)	35 ± 1.34	
3.	Gestational Age at Time of Diagnosis, In Weeks	17.6 ± 3.6	
4.	Mean Blood Glucose Level at the Time of Diagnosis (Mg/Dl)	123.5 ± 6.08	
		f	%
5.	Educational Status		
	Elementary	1	5.6
	High School	5	27.8
	College/University	9	42.9
	Postgraduate	6	33.3
6.	Employment Status		
	Employed	12	57.1
	Home Maker	9	42.9
7.	Economic Status		
	₹79,756-₹1,59,585	3	14.3
	₹59,795-₹79,755	8	38.1
	₹39,830-₹59,794	5	23.8
	₹23,870-₹39,829	5	23.8
8.	Religion		
	Hindu	14	66.7
	Muslim	5	22.2
	Christian	2	11.1
9.	Family History of Diabetes		
	Mother	9	42.9
	Father	5	23.8
	Any Other Family Members	7	33.3
10.	Current Treatment Approaches		
	Medical Nutrition Therapy	7	33.3
	Oral Hypoglycemic Agents	4	19.1
	Insulin Injections Only	2	9.5
	Combination Therapy	8	38.1

Thematic analysis revealed six main themes and several sub-themes that describe the participants’ lived experiences, perceptions, and health-seeking behaviours related to managing GDM. The findings offer rich insights into the emotional, informational, behavioural, and structural factors influencing health-seeking behaviour during pregnancy.

Theme 1: emotional response to diagnosis

Sub-theme 1: Fear and Anxiety

Most participants expressed feelings of fear and anxiety upon learning about their GDM diagnosis, particularly regarding the potential risks to their unborn child. “The first thing that came to my mind was, will my baby be okay? I was scared and confused", said one participant.

Sub-theme 2: Guilt and Self-Blame

Several women internalized the diagnosis and associated it with personal failure or poor lifestyle choices. One participant said, “I felt guilty. Maybe I didn’t eat properly, maybe I didn’t take care of myself enough”.

Sub-theme 3: Initial Denial or Minimization

A few participants reported initial disbelief or dismissal of the seriousness of the condition, particularly if they were asymptomatic. “I didn’t feel sick, so it was hard to believe it was something serious”, said one participant.

Theme 2: perceived adequacy of information and communication

Sub-theme 1: Inconsistent Information Delivery

Participants reported variability in the clarity, consistency, and usefulness of information received from healthcare providers. “Some doctors gave me detailed advice, but others just rushed me through. It was confusing,” said one participant.

Sub-theme 2: Need for Simplified and Culturally Sensitive Communication

Women, especially those from non-native language backgrounds, found it difficult to understand complex medical explanations. “I don’t understand medical terms. I needed someone to explain in simple words", said another participant.

Theme 3: expectations from healthcare providers

Sub-theme 1: Desire for Emotional Support and Empathy

Participants emphasized the importance of compassionate care beyond clinical advice. “I wanted someone to talk to, not just about the sugar levels but how I was feeling inside”, said a participant.

Sub-theme 2: Preference for Follow-Up and Continuity of Care

Several women expressed the need for regular check-ins and consistent care providers. “Every time I saw a new doctor, I had to explain everything again. I wished for the same person every time”, said another participant.

Sub-theme 3: Interest in Peer Support or Group Education

Participants believed interacting with others facing similar challenges could provide emotional relief and practical advice. Another participant said, “Talking to other pregnant women with the same condition would have made me feel less alone”.

Theme 4: positive health behaviour changes

Sub-theme 1: Improved Dietary Practices

Most women reported significant changes in their eating habits post-diagnosis, guided by dietary counseling. One participant said, “I stopped eating sweets and focused on balanced meals. I felt better, even more energetic.” 

Sub-theme 2: Increased Physical Activity

Some participants incorporated daily walks or light exercises into their routine. “I started walking every evening. It helped with my sugar and made me feel active", said a participant.

Sub-theme 3: Enhanced Health Awareness

For some women, the diagnosis served as a wake-up call that led to long-term lifestyle changes. One participant said, “Now I think more carefully about my health, even after the baby was born.” 

Theme 5: barriers to healthcare access

Sub-theme 1: Transportation and Geographic Limitations

Accessing specialist care was difficult for women living in rural or underserved areas. “I had to travel far to see a specialist. It was exhausting, especially in late pregnancy”, said one participant.

Sub-theme 2: Financial Constraints

Some women cited the cost of frequent appointments, dietary needs, and blood glucose monitoring supplies as burdensome. “The testing strips were expensive. I couldn’t afford them regularly”, said another participant.

Sub-theme 3: Language and Cultural Barriers

Non-native speakers reported difficulty navigating the healthcare system and understanding treatment recommendations. “I didn’t always understand what the doctor said, and I was too shy to ask again”, said one participant.

Theme 6: determinants of health-seeking behaviour

Sub-theme 1: Influence of Family and Social Support

Family members played a crucial role in either supporting or hindering women’s adherence to GDM management. One participant said, “My husband helped me follow the diet. Without him, I couldn’t have done it.” 

Sub-theme 2: Cultural Beliefs and Misconceptions

Some participants encountered traditional beliefs that downplayed the seriousness of GDM. “My mother-in-law said we didn’t have such problems in her day. She told me to eat what I wanted", said one participant.

Sub-theme 3: Competing Responsibilities

Childcare, work, and household duties made it difficult for women to prioritize their own health. One participant said, “I had two other kids at home. I couldn’t always go for checkups.” 

Sub-theme 4: Perceptions of Medical Interventions

Fear or mistrust of medications, especially insulin, deterred some women from following treatment plans. One participant said, “When they said insulin, I panicked. I thought it meant I was very sick.” 

A summary of the themes and sub-themes is presented in Table 2.

**Table 2:** Summary of Emergent Themes and Sub-themes

SL NO.	Themes	Sub-themes
1.	Emotional Response to Diagnosis	Fear and Anxiety; Guilt and Self-Blame; Denial or Minimization
2.	Perceived Adequacy of Information	Inconsistent Information: Need for Simplified and Culturally Sensitive Communication
3.	Expectations from Healthcare Providers	Desire for Empathy; Need for Continuity of Care; Peer Support
4.	Positive Health Behaviour Changes	Improved Diet; Increased Physical Activity; Long-term Health Awareness
5.	Barriers to Healthcare Access	Transportation/Geographic Limitations; Financial Constraints; Language/Cultural Barriers
6.	Determinants of Health-Seeking Behaviour	Family and Social Influence; Cultural Beliefs; Competing Responsibilities; Perceptions of Medical Interventions

## Discussion

This qualitative study explored how women diagnosed with GDM seek care, focusing on their emotional reactions, experiences with healthcare, behavioral changes, and the wider barriers they face. Overall, the findings suggest that women’s engagement with GDM management during pregnancy is shaped by a complex interaction of emotional, informational, and socio-cultural factors.

A GDM diagnosis often triggered immediate emotional distress, including fear, anxiety, guilt, and sometimes denial. These reactions were commonly tied to concerns about the baby’s health, feelings of self-blame, and the shock of facing a medical condition during what had seemed like a normal pregnancy. Similar patterns have been reported in previous studies, where women associated GDM with maternal inadequacy or feared negative outcomes for the unborn child [20, 21]. If these emotions are not properly addressed, they may delay or weaken active engagement in care.

Communication from healthcare providers emerged as another key factor. Women reported very different experiences with the information they received; some found it insufficient, while others felt it was too complex. This inconsistency often led to confusion and reduced confidence in medical advice. Earlier research has shown that clear, patient-centered communication improves adherence to GDM management strategies [22, 23]. The difficulties reported by non-native speakers in understanding medical terminology further underline the need for linguistically appropriate and culturally tailored education.

Women’s expectations of care went beyond clinical treatment. Participants wanted emotional support, continuity of care, and opportunities for peer interaction. Their preference for empathetic, holistic care is consistent with patient-centered models linked to better patient satisfaction and health outcomes [24]. Regular follow-up with the same healthcare provider was especially valued, suggesting that continuity helps build trust and supports ongoing care [25]. Group education and peer support were also seen as beneficial, as shared experiences helped reduce isolation and improve self-efficacy [26].

Many women also described making positive lifestyle changes, such as adjusting their diet, increasing physical activity, and becoming more conscious of their health. This suggests that a GDM diagnosis can function as a “teachable moment,” as described in health psychology literature [27]. However, the extent of these changes often depended on family support and the quality of counseling from healthcare professionals.

At the same time, participants identified several barriers to effective healthcare access, including geographic distance, financial pressure, language difficulties, and cultural beliefs. Women in rural areas particularly struggled to reach specialist services, reinforcing concerns about healthcare inequities in underserved regions [28]. Financial constraints further complicated care, with some women unable to afford frequent visits or essential supplies such as glucose testing strips. These findings point to the need for more equitable policies and subsidized support for pregnant women with GDM, particularly in low-resource settings [29].

Cultural beliefs and misconceptions also influenced how women perceived GDM and responded to treatment. Some participants reported that family members minimized the seriousness of the condition or discouraged adherence to dietary and medical advice. This supports previous research showing that cultural norms and family dynamics can either promote or undermine maternal health behaviors [30]. Fear and mistrust surrounding insulin therapy also emerged as a significant barrier, reflecting the stigma and misinformation that still surround pharmacological treatment during pregnancy [31].

Practical responsibilities, such as childcare and household duties, further limited women’s ability to seek care. This reflects broader gender-based inequalities, where women often place caregiving responsibilities ahead of their own health. Structural measures, including flexible appointment scheduling, childcare support, and community-based services, may help ease these burdens and improve engagement with care [32].

While this study provides valuable insights into the health-seeking behavior of women with GDM, certain limitations should be acknowledged. The data were collected from a single tertiary care hospital in Odisha, which may limit the generalizability of the findings to other settings. In addition, as the findings were based on self-reported experiences, the possibility of self-report bias cannot be ruled out. Nevertheless, the qualitative design enabled an in-depth exploration of the complex factors influencing health-seeking behavior among women with GDM.

## Conclusions

This study shows that women’s health-seeking behaviors in GDM are shaped by a combination of emotional, informational, socio-cultural, and health system-related factors. Psychological distress at the time of diagnosis, along with gaps in communication, can affect treatment adherence. In contrast, empathetic, consistent, and culturally appropriate care can improve women’s engagement with treatment and support services. The findings underline the need for holistic, context-sensitive interventions that go beyond biomedical management alone and address the psychosocial and structural factors affecting health-seeking behavior. Strengthening health systems through better communication, culturally relevant education, continuity of care, and accessible support services is therefore essential. Such measures may encourage greater maternal involvement, improve glycemic control, and lead to better outcomes for both mothers and their newborns. Future research should focus on designing and evaluating targeted interventions that bring these elements together to support women with GDM in different settings.

## Appendices

Appendix A

Interview Guide

1. Would you please share your experience when you were first time diagnosed with gestational diabetes?

2. How do you feel about the information you got from healthcare professionals after your diagnosis?

3. What kind of help and support do you wish to get from healthcare providers while dealing with gestational diabetes?

4. Can you share any positive outcomes or changes you noticed as a result of following the advice and guidance provided by healthcare professionals?

5. What challenges have you faced in accessing healthcare services and adhering to recommended health-seeking behaviors?

6. In your view, what are the main barriers or factors that make it difficult for you to seek or get help in managing gestational diabetes?
